# Spillover effects on infant caregiving and domestic work during the poriborton clean cookstove trial

**DOI:** 10.1371/journal.pone.0339949

**Published:** 2026-07-28

**Authors:** Md. Khobair Hossain, Neeloy Ashraful Alam, Elizabeth K. Kirkwood, Sajia Islam, Sk Masum Billah, Md. Rashidul Azad, S. M. Rokonuzzaman, Camille Raynes-Greenow

**Affiliations:** 1 Maternal and Child Health Division, icddr, b, Dhaka, Bangladesh; 2 Sydney School of Public Health, The University of Sydney, Camperdown, New South Wales, Australia; 3 House of Research and Development (HRD), Dhaka, Bangladesh; 4 Arnold School of Public Health, University of South Carolina, Columbia, South Carolina, United States of America; Omeo Kumar Das Institute of Social Change and Development, INDIA

## Abstract

Quality care during early childhood is essential for life-long health, well-being, and productivity. Recognizing the global importance of advancing early childhood development strategies, this study conducted in-depth, face-to-face interviews with women, their husbands, and mothers-in-law to explore perceived shifts in childcare roles among participants of Poriborton Trial, a randomized controlled trial of Liquefied Petroleum Gas (LPG) cookstoves. These interviews aimed to capture diverse perspectives on evolving caregiving dynamics and explored mothers’ perspectives. Qualitative data were systematically analyzed using thematic analysis following an iterative process of coding and theme development. We found that LPG stoves facilitated notable changes in caregiving. Firstly, LPG adoption reduced cooking time and decreased workload, which increased women’s time availability for childcare. Secondly, mothers reported a greater capacity to balance household tasks and childcare, fostering more frequent interaction with their children. Thirdly, the study identified enhanced childcare practices, with LPG use enabling women to be more responsive to their children's needs particularly during illness and to maintain closer physical proximity. The adoption of LPG stoves has multiple positive impacts on the childcare roles of breastfeeding women, empowering them to engage more fully in their children’s upbringing. Building on these qualitative insights into how LPG use may enhance childcare, future quantitative research is recommended to rigorously assess outcomes such as reductions in maternal stress, increased time availability for mothers, and changes in mother-child attachment attributable to LPG stove adoption.

## Introduction

In many low- and middle-income countries (LMICs), traditional gender roles remain deeply entrenched, with women primarily responsible for infant care and domestic work [1]. Care provided during the first years of life is critical for child development, influencing physical health, cognitive growth, and emotional well-being [2–4]. Quality early care during breastfeeding and early childhood can shape lifelong outcomes. However, these intensive caregiving phases coincide with significant demands on women’s time and energy, often creating a challenging environment for mothers [5,6].

In rural LMIC settings such as Bangladesh, new mothers experience challenges that are compounded by limited access to clean energy [7]. Most households rely on biomass fuels such as wood, crop residues, and dung for cooking. While these fuels are inexpensive and locally available, their use imposes a heavy burden on women. The process of collecting, cutting, drying, and storing biomass fuel is labor-intensive and time-consuming, often requiring hours of physical effort each week [6]. Cooking with biomass fuel also involves prolonged exposure to smoke and heat, which not only affects respiratory health but also contributes to fatigue and stress [8]. These cumulative demands reduce the time, and energy women can devote to childcare, potentially compromising the quality of care provided to infants [9,10].

The Poriborton trial was a community-based randomized controlled trial conducted in Sherpur district, Bangladesh, designed to evaluate the impact of liquefied petroleum gas (LPG) cooking compared to traditional biomass cooking on perinatal morbidity and mortality [11]. The trial was implemented across multiple rural communities and enrolled pregnant women and their households who were provided with LPG stoves and a continuous supply of LPG fuel as part of the intervention. The intervention was delivered during pregnancy [12]. While the primary focus of the trial was maternal and neonatal health outcomes, the introduction of LPG created the potential for broader changes in household practices, including caregiving roles.

Cooking with LPG offers several advantages over biomass fuel. It reduces cooking time, eliminates the need for fuel collection, and minimizes exposure to harmful smoke [7]. These changes can substantially decrease women’s physical workload and stress, freeing time for other activities. Time savings and reduced fatigue could positively influence caregiving practices, enabling mothers to engage more actively with their children, respond promptly to their needs, and maintain closer emotional and physical contact. Improved caregiving during infancy is associated with better health, nutrition, and developmental outcomes, making this an important area of inquiry [3].

Evidence from other LMIC contexts suggests that clean cooking interventions can have unintended or spillover effects beyond health, including improvements in gender equity, household dynamics, and psychosocial well-being [13]. However, these dimensions remain underexplored in Bangladesh, where cultural norms and economic constraints continue to shape women’s caregiving roles including domestic responsibilities. Understanding whether and how LPG adoption influences caregiving behaviors can provide important insights for designing interventions that address both health and social determinants of well-being.

This study therefore aims to explore the experiences of breastfeeding mothers who participated in the Poriborton trial. Specifically, it examines how the adoption of LPG stoves affected their ability to care for infants and manage domestic responsibilities. By focusing on caregiving practices, time allocation, and perceived stress, the study seeks to identify spillover effects of clean cooking interventions.

## Materials and methods

This qualitative study aimed to explore the unintended or spillover effects of LPG stove use on women’s caregiving roles in rural Bangladesh. The study focused on understanding how the adoption of LPG influenced women’s daily experiences and how these changes translated into shifts in caregiving practices and responsibilities. A systematic analytical approach was used to identify changes in women’s roles and caregiving practices, with emerging insights refined through discussions within the research team.

Pregnant women were enrolled into the trial at the time of pregnancy detection and received free LPG stoves and fuel from enrollment until their child reached 24 months of age. All mothers included in this qualitative study were therefore using LPG during the breastfeeding period at the time of interview.

Participants

We used purposive sampling to recruit participants from multiple villages within the Poriborton trial study area. A participant list was obtained from the trial implementation team, from which eligible participants were identified and approached. Eligible women were those previously enrolled in the intervention arm, who were breastfeeding mothers with an infant aged less than 24 months [13], and who were no longer receiving LPG, but may have continued to use it through their own funds.

To capture a broader understanding of caregiving dynamics within the household, we also included husbands and mothers-in-law of participating women, where available, as they are often key influencers in household decision-making in rural Bangladeshi contexts. Participation was not restricted to specific household structures; while many women lived with extended family members, including mothers-in-law and husbands, this was not a requirement for inclusion.

Participants were drawn from several villages, and efforts were made to include diversity in maternal experience, including both first-time and multiparous mothers. All participants were recruited through direct contact facilitated by the field team, and informed consent was obtained prior to participation.

### Data collection and analysis

We conducted in-depth interviews with participants in their households in settings that ensured privacy. All interviews were conducted individually and separately with mothers, husbands, and mothers-in-law to allow them to share their perspectives independently and to minimize potential influence from other family members.

Data were collected in November 2022 by a team of trained qualitative researchers (both male and female), who received training on the study objectives, interview techniques, probing methods, and ethical considerations, including informed consent and confidentiality. Each interview lasted approximately 40–60 minutes and was conducted in Bangla. Interviews were audio-recorded with participant’s consent.

The interview guide included open-ended questions to facilitate in-depth exploration of participants’ experiences and perceptions of LPG stove use, including its influence on daily routines, time use, childcare practices, and overall well-being. While this study focuses on caregiving-related outcomes, participants were also encouraged to discuss broader perceived benefits of LPG use, such as reduced smoke exposure and increased convenience. The full interview guide is provided in Appendix A.

Interviews were transcribed verbatim in Bengali using TRANSKRIPTOR [14] software and subsequently imported into ATLAS.ti [15] for coding and analysis.

We used an inductive thematic analysis approach following the framework described by Braun and Clarke to identify patterns and develop themes from data [16].

Two researchers independently coded the transcript and met regularly to compare and reconcile coding differences. The codes were then grouped into categories, which were further refined into themes through iterative discussions within the research team. Data were managed using atlas.ti software and backup copies were kept in password-protected computers accessed by the research team members only.

### Ethical considerations

This study was approved by the ethical review committee of icddr,b (PR-17103) and the Human Research Ethics Committee of the University of Sydney, Australia (0/2018/HE000717). Informed written consent was obtained from all participants before the interviews.

## Results

The study included 21 women and 10 men. Among the women, 11 were breastfeeding mothers enrolled in the Poriborton trial, and 10 were their mothers-in-laws. The 10 male participants were the husbands of breastfeeding mothers.

The findings are organized into three interconnected themes that describe how the adoption of LPG stoves influenced childcare practices among participating households. The first theme focuses on increased time availability resulting from reduced cooking burden and greater flexibility in managing household tasks. The second theme examines improvements in mothers’ caregiving capacity, driven by reductions in physical fatigue and psychological stress. Building on these enabling conditions, the third theme illustrates how gains in time and caregiver capacity were translated into increased opportunities for childcare and more responsive, attentive caregiving practices. Together, these themes reflect a sequential pathway through which LPG adoption shaped both the conditions and enactment of childcare within the household context (Table 1).

**Table 1:** Thematic framework of childcare changes linked to LPG stove use.

(A) Code	(B) Category	(C) Theme
Reduced cooking time Increased parallel activities Reduced cooking workload	Time Availability for Care Streamlined Household Tasks Reduced Domestic Burden	Theme 1: Increased time availability through reduced cooking burden
Decrease in physical exhaustion Decrease in mental stress of household roles	Improved Physical Well-being Improved mental Well-being	Theme 2: Enhanced Caregiver Capacity through reduced fatigue and stress
Increase in mother-child attachment Increase in caregiving during child illness Increase in night-time care Increase in quick responding to childcare needs	Enhanced Mother-Child Interaction Increased Responsiveness Proactive Caregiving	Theme 3: Increased caregiving opportunities through the translation of time and capacity gains

### Theme 1: Increased time availability through reduced cooking burden

Mother participants who were primary cooks reported increased time availability, which was primarily due to the efficiency of LPG stoves. Traditional clay stoves require continuous tending, including frequent feeding of fuel, maintaining the fire, and close monitoring to sustain combustion. These demands often require women to remain physically present near the stove, limiting their ability to engage in other tasks simultaneously. They consistently reported that LPG stoves substantially reduced cooking time, lessened their cooking workload, and created opportunities for engaging in parallel activities. These direct benefits translated into several key changes in their daily routines: increased time availability specifically for childcare, the ability to streamline other household tasks, and a subsequent reduction in their overall domestic burden. Consequently, the adoption of LPG technology appeared to provide mothers with additional time, a crucial resource which they can potentially allocate towards childcare responsibilities.

Every mother unanimously affirmed that this time-saving benefit has freed up time to engage more with their children. A mother described how she was able to take better care of her child after being an LPG stove user.

“*Normally, I have to do all the housework and bring up the children. Most of the time it gets difficult to take care of my child. But now after using the LPG stove, I save time and I get some (more) time to spend with my child*.” IDI-04

Another mother explained how the LPG stove reduced her cooking time by saying,

*“Now LPG use has saved my time …and I have the time for feeding my baby myself and taking leisurely walks with her. I can even enjoy precious moments of play with her.”* IDI-07

A mother-in-law shared how her daughter-in-law has effectively saved time managing household chores through the use of the LPG stove.

*“Before using LPG, my daughter-in-law was constantly rushed, spending hours on cooking. …Now, she has more time to care for her child, even holding them close whenever they cry.”* IDI-17

Several husbands shared that since their wives began using gas stoves, they could now complete their morning cooking and other tasks more time efficiently. One husband expressed,

*“…Using LPG has helped my wife prepare meals and even reheat food in the morning. Not only does she have time to bathe her baby and even hold her when she has time[...] and it feels good that she can take care of the baby along with all her chores.”* IDI-27

Participants expressed their ability to engage in parallel activities while cooking. These included simultaneously cutting vegetables while boiling water, attending to other household chores, or even pursuing personal interests. Both mothers-in-law and husbands observed these changes and the adoption of parallel activities, and included women taking baths, sweeping the yard, cleaning rooms tending to cattle, all while food was cooking on the LPG stove.

*“When I used to cook in clay stove, I had to stay with that one task for a long time, as a result of which the time goes by and the work does not progress. Now I can breastfeed my younger child, and also can sweep the rooms, leaving the food to cook on gas stove. During cooking now, I can perform additional 2-3 tasks simultaneously.” IDI-10*

A mother-in-law shared her observations about the multitasking abilities her daughter-in-law demonstrated while using the LPG stove.

*“My daughter in law had to allocate much time in front of the clay stove when she used to cook using dry leaves. She had to complete her daily tasks one by one since she could not leave the cooking place. But now gas stove has brought about a change, she sweeps the yard or takes a bath when she cooks.” IDI-20*

Women and mothers-in-law alike noted that these parallel activities included childcare responsibilities and various other household tasks.

### Theme 2: Enhanced caregiver capacity through reduced fatigue and stress

The second prominent theme emerging from the data reflects improvements in mothers’ caregiving capacity associated with reduced physical fatigue and psychological stress following the adoption of LPG stoves. Participants consistently described how the shift away from labor-intensive traditional cooking methods alleviated physical strain and reduced the burden of daily household tasks.

Several mothers directly contrasted the demanding nature of cooking with traditional clay stoves with the comparatively lower effort required when using LPG. Beyond physical relief, many also reported a decline in stress related to managing household responsibilities, contributing to an improvement in their physical and mental well-being. Husbands’ interviews often echoed this perspective, noting visible changes in mothers’ energy levels and emotional state.

### Decrease in physical exhaustion

Mothers expressed feeling less tired, less weak, and experiencing fewer bouts of dizziness after transitioning to LPG stoves. The reduced need for fuel collection, shorter cooking durations, and decreased physical exertion in the kitchen contributed to improved physical well-being.

*“I live with my mother-in-law and my sister in-law, but I do most of the household works. I used to collect dry leaves outside, slash solid fuels. Further, I spent long hours cooking on clay stove which often left me fatigued and sometimes even dizzy due to sitting longer in front of the stove. After starting cooking in gas stove, I do not have to sit in front of the stove at a stretch. Now I use gas stove, and I do not have to go outside to collect dry leaves or solid fuel which has greatly eased my physical burden. Now I get less tired and feel better physically.”* IDI-19

Moreover, many husbands and mothers-in-law reported improvements in mothers’ overall mood and daily functioning. A husband described the change in his wife following the reduction in her workload:

*“My wife often used to have a serious mood in the afternoon, and I could understand with her hard work throughout the day. It is good that now she uses a gas stove, many of her demanding tasks are no longer necessary. When I return home in the afternoon, I find her in a cheerful mood.”* IDI-15

### Decrease in mental stress of household roles

Mothers also described a reduction in mental stress, particularly related to the pressures of managing cooking and household responsibilities during busy periods of the day. The consistent availability of LPG and the reduced complexity of cooking helped alleviate anxiety and time-related pressures.

*“As a housewife I have to worry every time in my in-laws’ home wondering if I can cook quickly, provide breakfast for my children and above all, my husband. All these thoughts create mental stress for me. Now after being a gas stove user, I can ensure that my children can have breakfast before they go to school and my husband can start his day with a meal before going to work. So now my mental stress is eased.”* IDI-02

Several mothers-in-law also highlighted the reduction in morning stress, noting that women no longer faced uncertainty related to fuel availability or delays in meal preparation.

*“My daughter-in-law always tries to prepare breakfast for the whole family. But it was often difficult when she had to cook using dry leaves and straw. Sometimes there was no fuel, and she had to go out to collect it. Now, with the availability of LPG stoves, she prepares breakfast on time every day.”* IDI-26

A few husbands similarly observed that their wives appeared more relaxed and less pressured in managing daily household routines after adopting LPG.

### Theme 3: Increased caregiving opportunities through the translation of time and capacity gains

This theme demonstrates how gains in time availability and improvements in caregiver capacity created opportunities for mothers to engage more actively in childcare. Participants described how reduced time pressure and physical burden allowed them to translate these gains into more frequent, attentive, and responsive caregiving practices across different contexts.

### Increased mother-child closeness and interaction

Participants described that increased time flexibility and reduced work burden created new opportunities to remain physically close to their children while performing household tasks. This enabled more frequent holding, comforting, and interaction, contributing to greater mother-child closeness and engagement in daily routines.

One mother reflected on the contrast between her experiences with her children:

*“...My eldest baby wouldn't stop crying if I didn't cradle her in my arms. Unfortunately, I couldn't always hold her close because our old clay stove required frequent refueling, filling the kitchen with fumes. My youngest baby’s fate is better because we have a gas stove and cooking has become more manageable. Now I can hold my younger child on my lap while preparing meals. It's a remarkable feeling, as I couldn't provide the same comfort to my elder child in the past.”* IDI-13

Another mother described how improved flexibility allowed her to manage childcare alongside cooking:

*“Every morning at 11 a.m., like clockwork, the baby goes to sleep once more. I have discovered that lying down and breastfeeding her during this time helps her fall asleep faster and more peacefully. Previously, I could not leave the pots unattended for fear of burning, which often caused delays in putting the baby to bed, and the baby would cry in discomfort. Now I have a gas stove, I can control the cooking process. I reduce the flame and can now take some time to sleep beside the baby, and she seems content and calm without any insistence on her part.”* IDI-16

Breastfeeding emerged as an important domain where these opportunities became evident. Mothers described being able to breastfeed more frequently and without interruption as cooking tasks became more manageable. Reduced need for continuous monitoring of the stove allowed mothers to respond to feeding cues in a timely manner.

Family members also observed these changes in maternal engagement. As one mother-in-law noted:

*“Since receiving the gas stove, my daughter-in-law responds to the child much more quickly. She talks, sings, and interacts with the baby more than before.”* (IDI-29)

### Increased caregiving during child illness

Participants reported being better able to provide continuous and attentive care when children were ill. Previously, cooking demands often limit their ability to remain physically present with a sick child.

One mother explained:

*“When my first child was sick, I could not stay with them all the time because of household work like cooking, cleaning, maintaining cattle etc. But after getting the LPG stove for my second child, I now have the opportunity to stay close and take care of the child whenever she is ill.”* IDI-13

A mother-in-law similarly observed a change in caregiving behavior during illness:

*“Previously, when the baby was sick, I often had to take care of her because the mother was busy with household work. Now I see that she is able to hold the baby more often and stay with her during illness.”* IDI-17

### Improved night-time caregiving

Improved caregiving practices extended to night-time care, where mothers reported being more responsive and capable of attending to their children’s needs. Reduced workload and better energy management during the day enabled them to remain attentive at night.

One mother described:

*“If the baby defecates at night, it becomes very difficult to clean him. And if it is a winter night, I am very afraid that the baby will catch cold. Since we have a gas stove, heating water and cleaning the baby is much easier.”* IDI-06

A husband also reflected on this change:

*“My wife used to be exhausted after the whole day’s work and sometimes slept through the night even if the child cried. Since she started using a gas stove, she seems more attentive and is able to care for the child even at night.”* IDI-33

### Increased responsiveness to children’s needs

Another key improvement was mothers’ ability to respond more promptly and consistently to children’s immediate needs. Under traditional cooking conditions, safety concerns and time constraints often delayed responses.

One mother shared:

*“Before, if my child cried while I was cooking, I had to call out to him from the kitchen. I could not leave the stove because of the risk of fire. Now, with the gas stove, I can immediately go and pick up my child when he cries.”* IDI-11

Similarly, a mother-in-law observed:

*“Now, even while cooking, she picks up the baby whenever he cries and feeds him. She can manage both cooking and feeding the baby at the same time.”* IDI-20

Another participant highlighted this simultaneous caregiving ability:

*“When the baby cries, I can now pick the child up and continue cooking at the same time. With the traditional stove, this was not possible.”* IDI-45

## Discussion

This study explores women’s experiences and changes to their caregiving roles after receiving an LPG stove and fuel due to participating in the Poriborton trial in rural Bangladesh. Overall, the reduction in cooking time associated with LPG use emerged as a key driver of changes in caregiving practices. By decreasing the domestic workload burden related to household cooking, LPG use increased women’s available time and contributed to improvements in their well-being. These changes were experienced by the women and recognized by their immediate family members, including husbands and mothers-in-law. Collectively, these findings suggest that adopting clean cooking technologies may have benefits beyond reducing household air pollution, enabling more child-responsive care through increased time availability.

Existing evidence suggests that traditional stove use may impose a substantial time burden on women; however, empirical studies that objectively measure this time costs remain limited [17]. Similarly, changes in time use associated with cleaner cooking fuels have been reported in previous research [18,19]. In our study, compared to women’s experiences with biomass fuel, LPG use provided a consistent flame and higher heat, resulting in faster cooking that required less constant attention [12]. These factors allowed women to perform parallel activities and reduce time pressure, enabling them to be more attentive to their children. A study has shown that women who multitask during cooking can reduce overall cooking time [18]. In line with this, participants reported that time previously spent on demanding cooking tasks was reallocated toward childcare and interaction with their children [19].

As trial participants, households were provided with free LPG fuel until the birth of their child although some continued to purchase LPG after the trial had finished, which also reduced the need to collect and prepare solid fuels, further freeing up time. A systematic review on time savings associated with cleaner fuel adoption, although based on a limited number of studies, found reductions in fuel collection and cooking time [17]. While that review primarily emphasized income-generating potential, our study, which included breastfeeding mothers, found that this additional time was commonly used for infant care. Breastfeeding itself is a time-intensive activity, particularly in cases of exclusive breastfeeding [20].

Our findings are consistent with previous studies highlighting the importance of time management for breastfeeding mothers in providing care for their children [21]. By reducing the time required for cooking and enabling parallel task management, LPG use facilitated greater maternal availability for childcare.

In addition to time-related changes, increased caregiving capacity appeared to be supported by reductions in physical fatigue and mental stress. Participants reported decreased physical exhaustion and a reduction in stress related to their domestic responsibilities following the adoption of LPG stoves. The physically demanding process of collecting and preparing biomass fuel, combined with prolonged cooking, likely contributed to their prior fatigue.

Existing literature has documented the physical demands of rural women’s work [22,23], suggesting that labor-intensive activities contribute to fatigue and stress [15]. The reduction in these burdens, as reported by participants, may enhance women’s capacity to provide care. Consistent with quantitative findings that LPG use reduces the labor intensity of cooking [16], our results suggest that decreased fatigue may support mothers’ ability to engage more effectively in childcare.

Reduced fatigue and stress also appeared to improve women’s overall functioning within the household, allowing for greater engagement in caregiving activities [24]. This aligns with evidence suggesting that effective caregiving is closely linked to caregivers’ physical and psychological well-being [25,26]. By alleviating workload-related strain, LPG use may enable mothers to participate more actively in parenting.

Several studies further support these findings by highlighting the positive effects of cleaner cooking technologies on women’s well-being [27,28]. These improvements may, in turn, support their capacity to provide consistent and attentive care for their children. Consistent with findings from studies in India [29,30], time savings from LPG use appeared to increase mothers’ flexibility to attend to their children, indicating a potential reallocation of time toward caregiving.

Importantly, the increased time availability observed in this study was reflected not only in reduced workload but also in more frequent and attentive interactions between mothers and their children. Participants described being more physically present, responsive, and engaged in daily childcare activities, including feeding, soothing, and supervision. These changes represent an important shift from constrained caregiving toward more consistent and engaged parenting practices.

A substantial body of evidence suggests that early caregiver–child interactions, including responsive caregiving, verbal communication, and physical closeness, play a critical role in shaping children’s cognitive and socio-emotional development [31]. Increased maternal engagement creates opportunities for stimulation, learning, and emotional bonding, which are key components of early childhood development. Prior research has demonstrated that responsive and consistent caregiving is associated with improved language development, cognitive functioning, and emotional regulation in young children [32,33].

While this study did not directly measure child developmental outcomes, the observed improvements in maternal availability and caregiving practices suggest a potential pathway through which reductions in cooking-related burdens may indirectly influence child development. By enabling mothers to spend more time responsively engaged in caregiving, LPG stove adoption may contribute to the creation of more supportive early learning environments within the household.

An additional observation emerging from the data was that the flexibility afforded by LPG stoves allowed mothers to leave the cooking process unattended while engaging in other tasks, including childcare. While this facilitated increased caregiving opportunities, it may also introduce potential safety concerns, such as the risk of burns or cooking-related accidents if stoves are left unattended. Within the Poriborton trial, safety training was provided to all LPG users. Although this was not a primary focus of the study, these findings suggest that future clean cooking interventions may benefit from integrating safety guidance alongside efforts to promote timesaving and caregiving benefits.

### Strengths and limitations

A key strength of this study lies in its triangulation of findings through interviews with mothers, their husbands, and mothers-in-law. This multi-perspective approach facilitated a richer understanding of changes in practices following the adoption of LPG and helped validate mothers’ accounts by incorporating the viewpoints of their spouses and mothers-in-law. A potential limitation is response bias.

Participants received free LPG stoves from the project team and were aware that the researchers were affiliated with the project, which may have positively influenced their responses during interviews.

Further, we were not able to objectively assess parenting practices, breastfeeding behaviors, or time use, and therefore our findings are based on self-reported perceptions rather than direct measurement.

## Conclusion

The spill-over effect of participating in an LPG cookstove trial adds additional benefits for the participants. The LPG stoves reducing time spent cooking, and reduced the cookstove management, and fuel preparation associated with biomass cooking that enabled women to be more responsive to their infants. Participants also reported reduced fatigue and stress. These findings suggest that LPG stove use has the potential to alleviate the cooking-related burdens and support improved childcare. This evidence may inform future programs that integrate energy access with maternal and child health strategies, contributing to broader goals of women empowerment and sustainable development.

Investing in clean cooking may improve parental well-being and improve parent responsiveness to their child. While this study provided initial insights into potential pathways of improved childcare associated with LPG adoption, future quantitative research could further investigate these relationships. Specifically, quantitative studies are recommended to measure the extent of reduction in mental stress, the quantifiable increase in mothers’ time availability, and the degree of change in mother-child attachment following LPG stove adoption.

These findings highlight the potential for household-level interventions that reduce women’s time and labor burdens to generate downstream benefits for early childhood development through enhanced caregiving engagement.

## Supporting information

S1 Appendix Interview Guide for mothers, husbands and mother-in law. (DOCX)
