# Supplementary material for: Spillover effects on infant caregiving and domestic work during the poriborton clean cookstove trial
Source: PLoS One. 2026 Jul 28;21(7):e0339949. doi: 10.1371/journal.pone.0339949 (PMC13411896; doi:10.1371/journal.pone.0339949)
Supplement: S1 Appendix — (DOCX) [file pone.0339949.s001.docx]

**Appendix A:**

**Interview Guide (Mothers)**

1. Can you describe your daily routine before and after using the LPG stove?

2. In what ways has using the LPG stove changed the time you spend on cooking and other household tasks?

3. Can you explain whether using the LPG stove allows you to do other activities while cooking? If yes, what kind of activities?

4. How has the use of the LPG stove affected your overall workload and daily responsibilities?

5. Have you noticed any changes in how much time you are able to spend with your child since using the LPG stove? Please describe.

6. In what ways, if any, has using the LPG stove affected how you care for your child?

7. Can you describe any changes in your ability to respond to your child’s needs (e.g., feeding, soothing, attending to them when they cry)?

8. How has using the LPG stove influenced your ability to care for your child when they are sick?

9. Have there been any changes in your ability to care for your child at night since using the LPG stove?

10. How has using the LPG stove affected your physical well-being, such as tiredness or energy levels?

11. Have you experienced any changes in stress or mental pressure related to household work since using the LPG stove? Please describe.

12. In your opinion, what are the main benefits of using the LPG stove for you and your child?

13. Are there any challenges or concerns you have experienced while using the LPG stove?

**Interview Guide (Husbands)**

1. What changes have you observed in your household since the introduction of the LPG stove?

2. In your opinion, how has the LPG stove affected your wife’s cooking time and daily household workload?

3. Have you noticed whether your wife is able to perform other activities while cooking since using the LPG stove? If yes, please describe.

4. How has the use of the LPG stove affected the amount of time your wife spends with your child?

5. Have you observed any changes in how your wife interacts with your child (e.g., feeding, holding, responding to the child’s needs)?

6. In what ways, if any, has your wife’s care for the child changed since using the LPG stove?

7. How has the LPG stove influenced your wife’s ability to care for the child when the child is sick?

8. Have you noticed any changes in your wife’s ability to care for the child during the night?

9. In your view, has the LPG stove affected your wife’s physical condition, such as fatigue or tiredness? Please explain.

10. Have you observed any changes in your wife’s stress or mental pressure related to household work since using the LPG stove?

11. Overall, how do you think the LPG stove has affected your family life and childcare?

12. Are there any challenges or concerns you have observed related to the use of the LPG stove?

**Interview Guide (Mothers-in-law)**

1. What changes have you observed in your household since the introduction of the LPG stove?

2. In your opinion, how has the LPG stove affected your daughter-in-law’s cooking time and household workload?

3. Have you noticed whether your daughter-in-law is able to do other tasks while cooking since using the LPG stove? If yes, please describe.

4. How has the LPG stove affected the amount of time your daughter-in-law spends with her child?

5. Have you observed any changes in how your daughter-in-law cares for the child (e.g., feeding, holding, responding to the child’s needs)?

6. In what ways, if any, has her interaction with the child changed since using the LPG stove?

7. How has the LPG stove influenced her ability to care for the child when the child is sick?

8. Have you noticed any changes in how she cares for the child at night?

9. In your view, has using the LPG stove affected her physical condition, such as tiredness or energy levels? Please explain.

10. Have you observed any changes in her stress or mental pressure related to household responsibilities?

11. Overall, how do you think the LPG stove has affected childcare in your household?

12. Are there any challenges or concerns you have observed regarding the use of the LPG stove?
